# Context-Dependent Modulation of Corticomuscular Coherence in a Series of Motor Initiation and Maintenance of Voluntary Contractions

**DOI:** 10.1093/texcom/tgaa074

**Published:** 2020-10-07

**Authors:** Rina Suzuki, Junichi Ushiyama

**Affiliations:** Graduate School of Media and Governance, Keio University, Fujisawa 252-0882, Japan; Faculty of Environment and Information Studies, Keio University, Fujisawa 252-0882, Japan; Department of Rehabilitation Medicine, Keio University School of Medicine, Tokyo 160-8582, Japan

**Keywords:** beta-band oscillation, coherence, electroencephalogram, electromyogram, sensorimotor integration

## Abstract

For our precise motor control, we should consider “motor context,” which involves the flow from feedforward to feedback control. The present study focused on corticomuscular coherence (CMC) to physiologically evaluate how the sensorimotor integration is modulated in a series of movements depending on the motor context. We evaluated CMC between electroencephalograms over the sensorimotor cortex and rectified electromyograms from the tibialis anterior muscle during intermittent contractions with 2 contraction intensities in 4 experiments. Although sustained contractions with weak-to-moderate intensities led to no difference in CMC between intensities, intermittent ballistic-and-hold contractions with 2 intensities (10% and 15% or 25% of the maximal voluntary contraction, MVC) presented in a randomized order resulted in greater magnitude of CMC for the weaker intensity. Moreover, the relative amount of initial error was larger for trials with 10% of MVC, which indicated that initial motor output was inaccurate during weaker contractions. However, this significant difference in CMC vanished in the absence of trial randomization or the application of intermittent ramp-and-hold contractions with slower torque developments. Overall, CMC appears to be modulated context-dependently and is especially enhanced when active sensorimotor integration is required in feedback control periods because of the complexity and inaccuracy of preceding motor control.

## Introduction

Recognizing the current state of movements is essential for maintaining precise motor control. To achieve that, a feedback control system in the human nervous system is widely considered to play an important role in integrating sensory information into the generation of motor commands, enabling ongoing improvement of performance. This phenomenon is referred to as sensorimotor integration and is known to contribute to sophisticated movements.

Coherent oscillations between the sensorimotor cortex and contracting muscles, known as corticomuscular coherence (CMC), can be evaluated as a physiological indicator of sensorimotor integration. CMC was first observed in the 1990s in both monkeys (Baker et al. 1997) and humans (Conway et al. 1995), and it was initially considered to reflect top-down control from the cortex to the peripheral muscles (Conway et al. 1995; Mima et al. 2000). Subsequent studies revealed that attenuation of somatosensory feedback could modulate CMC (Pohja and Salenius 2003; Riddle and Baker 2005) and indicated that cortical oscillations could be encoded from peripheral sites (Baker 2006). In light of these findings, CMC is currently believed to include bidirectional interactions between the cortex and periphery. Although its functional role is still under debate, it is assumed to reflect the extent of sensorimotor integration that has been continuously updated in a series of movements.

However, in previous CMC studies, the “motor context,” which involves a flow from feedforward to feedback control, was not fully considered within the experimental paradigms. At the initial stage of motor control, feedforward control always occurs which does not refer to somatosensory feedback. Due to the sensorimotor delay, motor initiation would be achieved by feedforward control followed by feedback control. On the other hand, CMC analyses have mostly been performed using stable tonic isometric contractions and have focused solely on feedback control. However, the accuracy of initial motor output determined by both feedforward and feedback control can vary, even in similar motor tasks, and thus intervene in the subsequent maintenance period of motor output by pure feedback control. From this perspective, previous studies examining functional roles played by CMC in motor control have not always accounted for the motor context of the participants. The motor control strategy should vary depending on what the participants are required to do, for instance, whether they are asked to simply produce a stable motor output or are actively required to correct their motor error. In the field of computational neuroscience, the signal-dependent noise theory (Harris and Wolpert 1998) proposes that feedback control is actively required when large motor error is induced by the magnified motor commands necessary for rapid movement. Consequently, the functional role of CMC should be discussed in depth regarding the series of motor events within a movement.

Here, we examined how CMC is modulated in a series of movements that included a flow from initiation to maintenance of the motor output. Some previous studies using simple tonic contraction tasks reported that the magnitude of CMC was not affected by the contraction level within a weak-to-moderate intensity range (Brown et al. 1998; Mima et al. 1999; Ushiyama et al. 2012). In the present study, we mainly explored whether this no change in CMC would also occur during intermittent ballistic-and-hold contractions where participants were required to repeat rapid motor output followed by stabilization of their movements. When we tested this hypothesis, we unexpectedly found that the magnitude of CMC was stronger in the weak contractions with greater inaccuracy of the initial torque output. Subsequently, we carried out 3 additional experiments in which we changed the parameters of the intermittent contraction tasks, such as the contraction intensity, trial randomization, and torque development speed, to see changes in CMC depending on the motor context of the participants.

## Materials and Methods

### Ethical Approval

The experiments were carried out in accordance with the Declaration of Helsinki and were approved by the ethical committee of the Faculty of Policy Management, Faculty of Environment and Information Studies, and Graduate School of Media and Governance, Keio University (receipt number 167). All participants received a detailed explanation of the experiment and provided informed consent prior to participation.

### Participants

Participants included 17, 15, 13, and 12 healthy young adults who completed experiments 1, 2, 3, and 4, respectively (see Experimental Protocol for further information). None of the participants reported a history of neuromuscular or musculoskeletal disorders.

### Recordings

Participants sat comfortably on a chair with an ankle dynamometer fitted to their right foot via 2 straps. Their right knee was set at 60° flexion from full extension. A monitor showing visual feedback was positioned 2 m in front of them at eye level. Before the experiment, we recorded the ankle dorsiflexion torque of each participant during maximal voluntary contraction (MVC) after sufficient practice.

Scalp electroencephalograms (EEGs) were recorded from 5 scalp positions (Cz, C1, C2, FCz, and CPz) over the sensorimotor area according to the International 10–20 system. 5 Ag/AgCl electrodes with a diameter of 18 mm (g.LADYbirdPASSIVE 1035, Guger Technologies, Graz, Austria) were affixed to an EEG cap (g.GAMMAcap 1027; Guger Technologies, Graz, Austria) covering the area representing the foot region. Reference and ground electrodes were placed on the left and right earlobes, respectively. Surface electromyograms (EMGs) were recorded from the tibialis anterior (TA) muscle and the soleus (SOL) muscle by placing passive bipolar Ag/AgCl electrodes with a diameter of 10 mm placed on the muscle belly, leaving 30 mm between the proximal and distal electrodes. We decided to record from the TA because the distal lower limb muscles have been found to show prominent CMC (Ushiyama et al. 2010; Gwin and Ferris 2012) and are important for daily movements such as locomotion (Petersen et al. 2012) and posture (Geertsen et al. 2013; Spedden et al. 2018; Spedden et al. 2019). The SOL was recorded to ensure that the participants were not co-contracting the antagonist of the TA. The torque signal that emerged during ankle dorsiflexion was recorded via an ankle dynamometer (TCF100N; Takei Scientific Instruments Co., Ltd.). EEGs and EMGs were amplified and band-passed (EEG, 0.5–200 Hz; EMG, 5–500 Hz) via an analog biosignal amplifier (g.BSamp 0201A; Guger Technologies). The torque signal was low-pass filtered using a second-order Butterworth filter at a cutoff frequency of 50 Hz and amplified by an amplifier (DPM-711B; Kyowa Electronic Instruments Co., Ltd.). Analog EEG, EMG, and torque signals were converted into digital signals by an analog-to-digital converter (NI USB-6212 BNC, National Instruments) at a sample rate of 1000 Hz, controlled by a data-logger program designed using MATLAB software (The MathWorks, Inc.).

### Experimental Protocol

Since the magnitude of CMC varies among individuals (Ushiyama et al. 2011b), we included participants who showed significant $\beta$-band (15–35 Hz) CMC in the experiment and analyses. In Experiment 1, we screened participants using a sustained isometric contraction task and rejected the participants who did not show significant CMC. We then conducted Experiments 2–4 on the basis of the results of Experiment 1. The order of Experiments 2–4 was randomized for each participant who agreed to participate more than once. For these experiments, we mainly recruited participants who showed significant CMC in Experiment 1.

#### Experiment 1

Seventeen healthy young adults (8 men and 9 women, aged 19–25 years) participated in Experiment 1, which examined how the motor requirement to the participants would alter their CMC. Because many CMC studies adopt a tonic contraction period for analyses (e.g., Kamp et al. 2013; Matsuya et al. 2017; Spedden et al. 2018), we compared the magnitude of CMC obtained using a conventional protocol (i.e., sustained contractions) with that from the intermittent task performed via ballistic-and-hold contractions. In the sustained task, the participants performed a simple tonic contraction and were required to track the visual feedback in a static manner. Conversely, they needed to actively correct initial error in the intermittent task, where they were asked to exert torque as quickly as possible, which resulted in substantial overshooting with respect to the target. We hypothesized that for the trials in the intermittent task that require active error correction, sensorimotor integration would be enhanced, resulting in increased CMC.

The motor task was divided into 2 sections—the sustained task and the intermittent task. For the sustained task, the participants performed static isometric ankle dorsiflexion for more than 65 s at 10% and 15% of MVC in a random order. Visual feedback was given on the screen, where a vertical blue line indicated the target torque and a red square marker represented the torque level produced by the participant. They were asked to bring the red marker to the blue line and hold it there as precisely as possible except for during the resting period. The sustained task was performed once for each contraction intensity. For the intermittent task, the participants repeated intermittent ballistic-and-hold contractions at 10% and 15% of MVC. For the first 7 s of each trial, the participants could move freely and change their posture (“Rest” period). This was followed by a 2-s “Relax” period in which they were asked to stay still. Then, an auditory stimulus was presented 3 times with an interval of 1 s (“Ready” period) to signal the participants to get ready for the upcoming trial. After that, they were asked to dorsiflex their right ankle as quickly as possible and track the visual feedback as precisely as they could for 6 s (“Task” period). The trial ended with a 2-s Relax period. This flow occurred for each trial, and each set consisted of 12 trials with 2 contraction intensities (6 trials each) presented in random order. Each participant completed 10 sets. During the intermittent task, interval periods 5–10 min long were presented at least once for every 2 sets to avoid task-relevant fatigue.

#### Experiment 2

Fifteen healthy young adults (7 men and 8 women, aged 18–25 years) participated in Experiment 2. In this experiment, we examined the effect of contraction level by increasing the difference between the 2 intensities. In Experiment 1, we found a significant difference in CMC between the 2 intensities in the intermittent task. Since we adopted 2 close intensities in Experiment 1, that is, 10% and 15% of MVC, we sought to examine whether this closeness contributed to the obtained result. We hypothesized that the proximity of the 2 intensities might have contaminated recognition of the contraction intensity, leading to increased task difficulty. To test this, we set the intensity difference to a level that the participants could clearly recognize and determined whether the significant difference in CMC between the contraction intensities would be diminished or consistent with respect to the findings from Experiment 1. The experimental protocol was similar to that in Experiment 1 and included the sustained task and the intermittent task, although the contraction intensities were set at 10% and 25% of MVC.

#### Experiment 3

Thirteen healthy young adults (8 men and 5 women, aged 19–25 years) participated in Experiment 3. In Experiments 1 and 2, we examined CMC during the intermittent task with the presentation order of the 2 intensities randomized to prevent the participants from become familiarized with the motor task. However, the results from these 2 experiments may have differed if the presentation order had not been randomized. In Experiment 3, we used a simplified motor task. Thus, we expected that, as in Experiment 1, a significant difference in CMC according to differences in contraction intensity might not be observed. In this experiment, the participants performed the intermittent task with 10% and 15% of MVC, respectively, without trial randomization. They repeated ballistic-and-hold isometric ankle dorsiflexion with 12 trials for each set and 5 sets for each contraction intensity. After 5 sets with 1 intensity were complete, the participants performed a practice set with the other intensity and then completed the other 5 sets. The intensity order (first half vs. latter half) was randomly determined for each participant.

#### Experiment 4

Twelve healthy young adults (7 men and 5 women, aged 18–25 years) participated in Experiment 4. Unlike the other 3 experiments, the participants performed intermittent ramp-and-hold contraction with 10% and 15% of MVC, where the ramp phase lasted for 1 s. During the ballistic-and-hold contraction, rapid torque development would lead to large motor error as part of the speed-accuracy trade-off described in Fitts’ Law (Fitts and Peterson 1964). Therefore, we expected the participants to correct the initial error during every trial in the intermittent task in Experiments 1–3. By allowing the participants to slowly contract their muscles in Experiment 4, we sought to examine whether the absence of large initial errors would affect the CMC compared with that in the task that involved ballistic-and-hold contractions. If no significant differences were observed between the intensities in this intermittent ramp-and-hold contraction task, we could conclude that functional role of CMC differs depending on the necessity of initial error correction. The contraction intensities were randomized in a similar manner to that in Experiment 1 (total 10 sets, 120 trials). The participants were required to track the visual feedback as precisely as possible throughout the motor task and were prohibited from rapidly developing torque during the movement.

### Data Analyses

**Figure 1:**
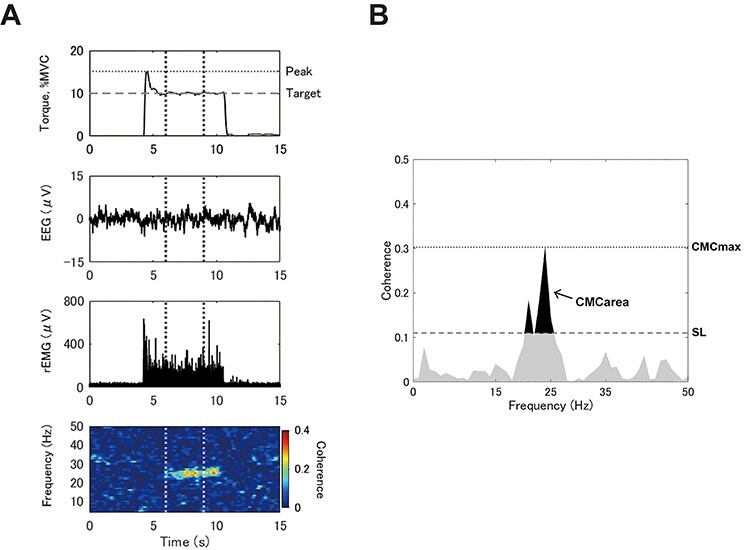
(*A*) Typical examples of a raw torque signal, a raw electroencephalogram (EEG) over the vertex of the primary motor cortex, and a rectified electromyogram (rEMG) recorded during the intermittent task (10% of the maximal voluntary contraction, MVC). The bottom row shows an example of a time–frequency map of coherence between EEG and rEMG. Note that the time–frequency map was generated from data from 60 trials and does not represent only 1 trial. The difference between the target and peak value of the exerted torque is defined as “initial error”. The analyzed section, which was 3-s long, is presented between the vertical dotted lines. (*B*) An example of the coherence spectrum between EEG and rEMG during isometric ankle dorsiflexion. The maximal value of corticomuscular coherence (CMC) was defined as CMCmax. Statistically significant values are above the significance level (SL) within the β-band (15–35 Hz), and areas shaded in black represent CMCarea.

EEG signals were Laplacian-filtered at Cz to emphasize its signal by subtracting the averaged potentials from the surrounding channels such as C1, C2, FCz, and CPz from Cz (Hjorth 1975) and low-pass filtered by a second-order Butterworth filter at 48 Hz. EMG signals were all rectified. EMG rectification is considered suitable for CMC analysis as it emphasizes grouped discharge and extracts the oscillatory envelope (Myers et al. 2003; Ward et al. 2013; Dakin et al. 2014). For the sustained task, a continuous 60-s period with few mechanical artifacts caused by postural change and/or eye blinking was extracted from the EEG, EMG, and torque signals for each contraction intensity. For the intermittent task, a 3-s period immediately after the exerted torque became relatively stable was extracted for each trial (Fig. 1*A*). When the initial error was induced by the ballistic-and-hold contraction, the exerted torque will follow a flow, including overshoot, undershoot, fluctuation, and convergence with respect to the target. In the present study, we focused on CMC during this fluctuation period. For data analysis, the moment when the exerted torque intersected the target, a third time was considered to be the beginning of the fluctuation period in which the prominent overshoot/undershoot at the initial stage of torque development is corrected. The extracted 3-s periods for each intensity level from the 60 trials were combined for further analyses.

To calculate coherence values, raw EEG and rectified EMG signals from the TA were segmented into 1-s data windows with a total of 60 and 180 data epochs in the sustained task and intermittent task, respectively, without overlap. The Hanning window was applied to each data epoch to reduce spectral leakage (Farmer et al. 1993; Baker et al. 1997; Gross et al. 2000), and coherence between the 2 signals was calculated from the following equation (Halliday et al. 1995):

(1) \begin{equation*} \left|{\boldsymbol{C}}_{\boldsymbol{xy}}\left(\boldsymbol{f}\right)\right|=\frac{{\left|\overline{{\boldsymbol{P}}_{\boldsymbol{xy}}\left(\boldsymbol{f}\right)}\right|}^{\mathbf{2}}}{\overline{{\boldsymbol{P}}_{\boldsymbol{xx}}\left(\boldsymbol{f}\right)}\cdot \overline{{\boldsymbol{P}}_{\boldsymbol{yy}}\left(\boldsymbol{f}\right)}}, \end{equation*}

where $\overline{P_{xx}(f)}$ and $\overline{P_{yy}(f)}$ are the averaged power spectral density (PSD) of the EEG and rectified EMG signals at a given frequency $(f)$, respectively. $\overline{P_{xy}(f)}$ is the averaged cross-PSD between those 2 signals. The coherence value ${C}_{xy}(f)$ ranges from 0 to 1, where 1 suggests a complete correlation. A confidence limit of 95% was determined as the significance level (SL) according to previous studies (Halliday et al. 1995). To eliminate the possibility that the coherence values were judged to be significant due to statistical errors, a Bonferroni correction was applied to the equation with a 95% confidence limit to carry out multiple comparisons. This is in accordance with previous studies (Ushiyama et al. 2011a; Gwin and Ferris 2012; Petersen et al. 2012) and used the following equation:

(2) \begin{equation*} {\displaystyle \begin{array}{c}\mathbf{SL}\left(\boldsymbol{\alpha} \right)=\mathbf{1}-{\left[\dfrac{\mathbf{1}}{\boldsymbol{N}+\mathbf{1}}\cdot \left(\mathbf{1}-\dfrac{\boldsymbol{\alpha}}{\mathbf{1}\mathbf{00}}\right)\right]}^{\frac{\mathbf{1}}{\boldsymbol{L}}-\mathbf{1}},\end{array}} \end{equation*}

where *L* is the number of data segments (i.e., 60 for the sustained task, 180 for the intermittent task with 3 s × 60 trials), *N* is the number of frequency bins (i.e., 48; between 3 and 50 Hz), and $\alpha$ is the *P* value, which had a confidence limit of 95% in the present study. The SL was 0.1099 for the sustained task and 0.0376 for the intermittent task. Only the coherence values that exceeded the SL were interpreted as significant. We quantitatively evaluated the maximal value and the sum of significant CMC within the $\beta$-band (15–35 Hz) and defined these as CMCmax and CMCarea, respectively (Fig. 1*B*).

To evaluate the steadiness of the exerted torque during isometric contraction, the coefficient of variation (CV) was calculated as follows:

(3) \begin{equation*} {\displaystyle \begin{array}{c}\mathbf{CV}=\dfrac{\mathbf{standard}\ \mathbf{deviation}}{\mathbf{mean}}\times \mathbf{100}\%,\end{array}} \end{equation*}

where a larger torque CV suggests greater performance instability. The data used for analysis was different for each task. For the sustained task, we extracted a continuous 60-s period for analyses. For the intermittent task, the torque CV from each trial was first calculated (3 s each) and then the entire average across trials was determined as the final value.

For the intermittent task, we evaluated the magnitude of the initial error in each trial by differentiating the torque during the ballistic-and-hold contraction. We looked for the peak of the torque right after the moment where the maximal derivative was found and subtracted the target value to find the initial error size. This procedure was repeated for all 60 trials for each intensity level. Then, the mean and standard deviation (SD) values were calculated within each participant. While the average initial error shows the deviation from the target, the SD of the initial error could be used to evaluate the variation in initial motor output such that a smaller SD indicates more stability. Considering the signal-dependent noise theory (Harris and Wolpert 1998), we expected to obtain larger values for stronger intensities. Therefore, apart from the absolute value of the percentage of the MVC, the relative value with respect to the target was also calculated by dividing the absolute value by the contraction intensities. This was defined as the “initial error rate,” where zero indicated that the torque level was similar to the target intensity. By finding the relative value, we could compare the extent of error not just within 1 intensity but also between 2 different intensities as it could tell to what extent the deviation was substantial for that target level specifically.

### Statistics

Statistical analyses were carried out using SPSS Statistics version 25 (IBM SPSS Inc.). To examine the potential differences in CMCmax, CMCarea, and torque CV according to contraction intensity, we performed paired *t*-tests with the null hypothesis that each dependent variable would be constant across the factors. A similar analysis was performed for the initial error size and initial error rate in Experiments 1–3. *P* values of 0.05 were adopted for all the statistical analyses to signify statistical significance.

## Results

In the present study, we adopted CMCmax and CMCarea as indicators of CMC. However, the figures only focus on CMCmax as a quantitative measure of CMC because the results and statistical significance for CMCmax and CMCarea were similar in all experiments (see the following sections for details regarding each experiment).

### Experiment 1

**Figure 2:**
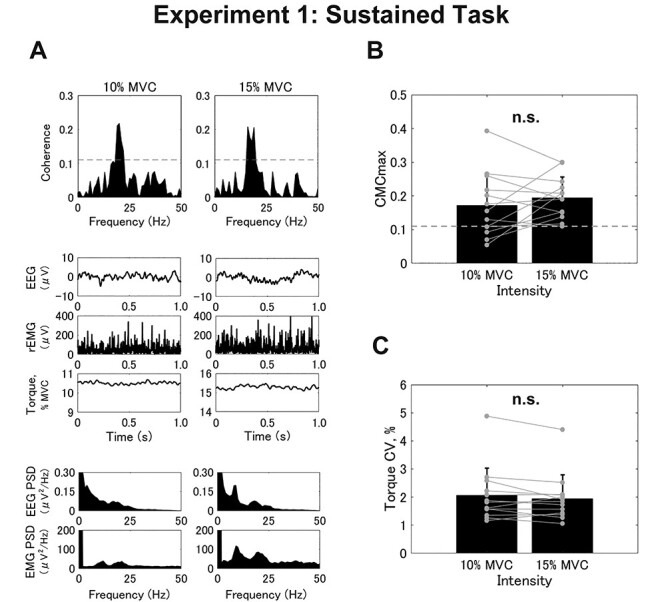
Results from the sustained task (Experiment 1). (*A*) Typical examples of coherence spectra between EEG and rEMG (top row), raw EEG signals, rEMG, and raw exerted torque (middle row), and power spectra for EEG and rEMG (bottom row). (*B* and *C*) Group data (mean ± SD) for CMCmax (*B*) and torque coefficient of variation (CV) (*C*). Black bars represent the average values for each contraction intensity. Black lines on these bars show standard deviations (SDs). The gray circles that are connected with a line represent data points from each participant. n.s. indicates no significance.

In the sustained task, 13 out of 17 participants showed statistically significant CMC. This was the case for 12 participants in the intermittent task. All participants whose CMCmax exceeded the SL showed the peak frequency within the $\beta$-band (Sustained: 10%, 21.875 ± 3.563 Hz; Sustained: 15%, 23.000 ± 5.155 Hz; Intermittent: 10%, 24.636 ± 4.632 Hz; Intermittent: 15%, 24.909 ± 3.673 Hz). Figures 2*A* and 4*A* represent typical examples of data from the sustained task and intermittent task, respectively. In the sustained task, CMC spectra, raw EEG, rectified EMG, and torque signals did not differ between the 2 intensities. Although the rectified EMG signal was larger in amplitude for the trial with 15% of MVC, which was expected, as greater muscular activity was required compared with the trial with 10% of MVC, there were no other prominent differences in EMG PSD between the 2 intensities (Fig. 2*A*). In the intermittent task, the $\beta$-band oscillation was relatively prominent in the rectified EMG and torque signals from the trial with 10% of MVC compared with those with 15% of MVC. Moreover, CMCmax was clearly larger in trials with 10% compared with 15% of MVC (Fig. 4*A*).

For the group data (mean ± SD) from the sustained task, we found no significant differences between the trials with 10% and 15% of MVC for either CMCmax (10%, 0.172 ± 0.095; 15%, 0.195 ± 0.061; *t* = −1.038, *df* = 12, *P* = 0.320; Fig. 2*B*) or CMCarea (10%, 0.499 ± 0.665; 15%, 0.694 ± 0.483; *t* = −1.933, *df* = 12, *P* = 0.077). This is consistent with previous studies that reported no significant differences in CMC magnitudes among weak-to-moderate contraction levels (i.e., 10–40% of MVC) during sustained contraction (Brown et al. 1998; Mima et al. 1999; Ushiyama et al. 2012). The torque CV was also not significantly different between the 2 intensities (10%, 2.055 ± 0.974%; 15%, 1.947 ± 0.838%; *t* = 1.376, *df* = 12, *P* = 0.194; Fig. 2*C*).

**Figure 3:**
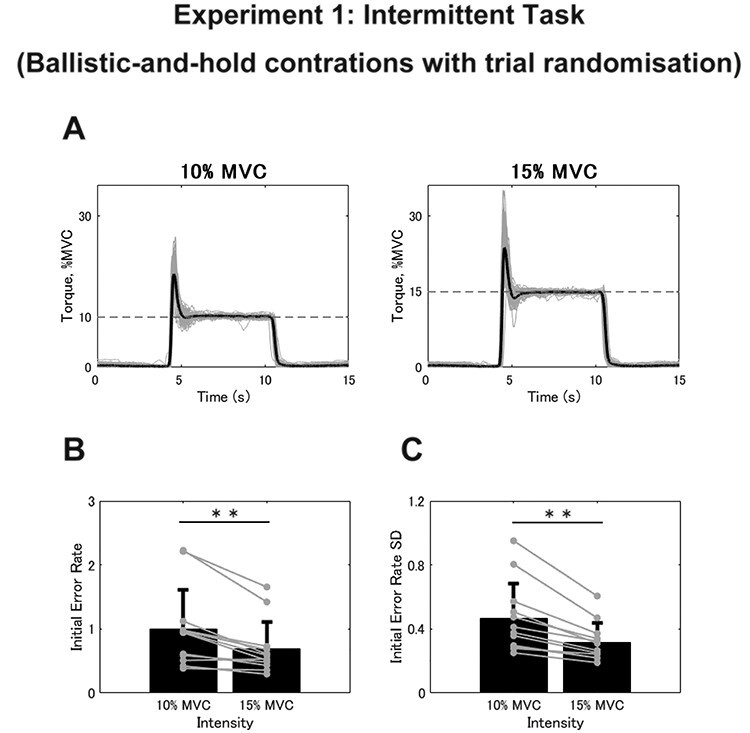
(*A*) Typical examples of torque signals during the intermittent task in Experiment 1. Gray plots represent the torque for the 60 trials and the black plot represents their average. Dashed lines represent the target intensities. (*B* and *C*) Group data for the individual mean initial error rate (*B*) and SD (*C*) calculated from 60 trials. Black bars represent the average values for each contraction intensity. Black lines on these bars show SDs. The gray circles that are connected with a line represent the data points from each participant. Significant differences between the contraction intensities are denoted as follows: ^*^P < 0.05, ^*^^*^P < 0.01.

**Figure 4:**
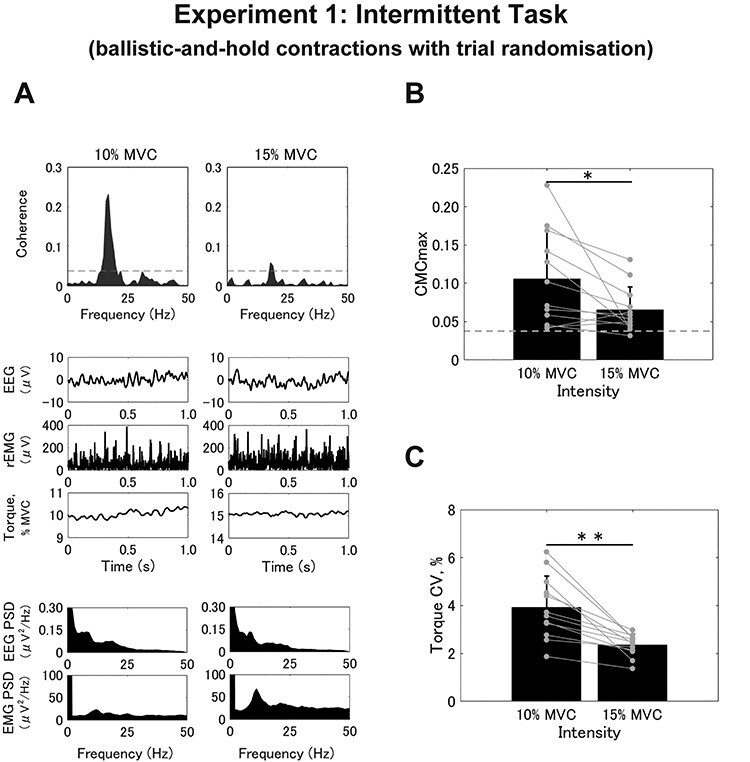
Results from the intermittent task (Experiment 1). (*A*) Typical examples of coherence spectra between EEG and rEMG (top row), raw EEG signals, rEMG, and raw exerted torque (middle row), and power spectra for EEG and rEMG (bottom row). (*B* and *C*) Group data for CMCmax (*B*) and torque CV (*C*). Black bars represent the average values for each contraction intensity. Black lines on these bars show SDs. The gray circles that are connected with a line represent the data points from each participant. Significant differences between the contraction intensities are denoted as follows: ^*^P < 0.05, ^*^^*^P < 0.01.

For the intermittent task, we first determined the initial error value for each trial to evaluate the accuracy of initial motor output. Then, the averages and SDs from the 60 trials were calculated for each intensity value. The representative example of the torque signals is shown in Figure 3*A*. In terms of absolute values, there were no differences in either the initial error (10%, 9.941 ± 6.224% MVC; 15%, 10.356 ± 6.272% MVC; *t* = −0.913, *df* = 11, *P* = 0.381) or SD (10%, 4.617 ± 2.187% MVC; 15%, 4.761 ± 1.799% MVC; *t* = −0.907, *df* = 11, *P* = 0.384). Surprisingly, however, when the initial error value was converted into a relative value with respect to the target (initial error rate), we found significantly larger values for 10% of MVC compared with 15% of MVC for both the initial error rate (10%, 0.994 ± 0.622; 15%, 0.690 ± 0.418; *t* = 4.359, *df* = 11, *P* = 0.001; Fig. 3*B*) and the SD (10%, 0.462 ± 0.219; 15%, 0.317 ± 0.120; *t* = 4.817, *df* = 11, *P* = 0.001; Fig. 3*C*). This indicates that a weaker intensity could result in relatively greater motor error and variability per trial. In terms of indicators that reflect the behavior of feedback control, we observed a significant difference between the 2 intensities for CMCmax (10%, 0.106 ± 0.062; 15%, 0.065 ± 0.298; *t* = 2.534, *df* = 11, *P* = 0.028; Fig. 4*B*) and CMCarea (10%, 0.414 ± 0.345; 15%, 0.212 ± 0.216; *t* = 2.613, *df* = 11, *P* = 0.024). Moreover, the torque CV was also greater in the trials with 10% compared with 15% of MVC (10%, 3.919 ± 1.315%; 15%, 2.344 ± 0.459%; *t* = 4.679, *df* = 11, *P* = 0.001; Fig. 4*C*). In summary, 10% of MVC resulted in a larger initial error rate than 15% of MVC, also with larger magnitudes of CMC and torque CV in the intermittent task, unlike the results from the sustained task.

### Experiment 2

**Figure 5:**
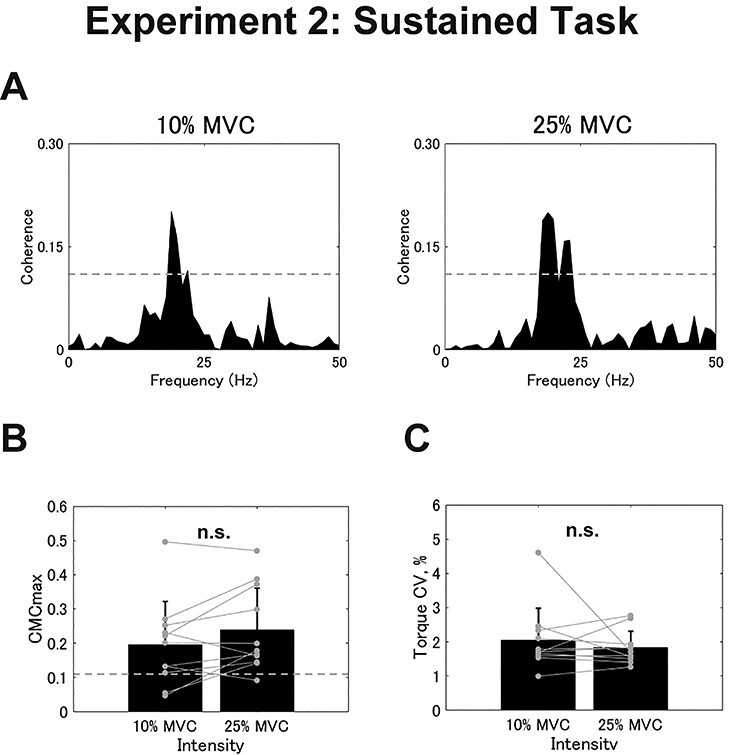
Results from the sustained task in Experiment 2. (*A*) Typical examples of coherence spectra between EEG and rEMG for 10% and 25% of MVC. The dashed line represents the SL. (*B* and *C*) Group data for CMCmax (*B*) and torque CV (*C*). Black bars represent the average values for each contraction intensity. Black lines on these bars show SDs. The gray circles that are connected with a line represent the data points from each participant. n.s. indicates no significance.

In Experiment 2, 11 out of the 15 participants showed significant CMC in the sustained task and intermittent task. All participants whose CMCmax exceeded the SL showed the peak frequency within the $\beta$-band (Sustained: 10%, 23.875 ± 3.643 Hz; Sustained: 25%, 24.250 ± 3.240 Hz; Intermittent: 10%, 27.500 ± 2.429 Hz; Intermittent: 25%, 29.667 ± 1.862 Hz). Figure 5*A* represents a typical example of the CMC spectra, where no remarkable differences were observed between the 2 intensities, that is, 10% and 25% of MVC. As in Experiment 1, the sustained task resulted in no significant differences between the 2 intensities for either CMCmax (10%, 0.196 ± 0.125; 25%, 0.238 ± 1.123; *t* = −1.918, *df* = 10, *P* = 0.084; Fig. 5*B*) or CMCarea (10%, 0.665 ± 0.757; 25%, 1.018 ± 1.132; *t* = −2.059, *df* = 10, *P* = 0.066). This pattern was similar for the torque CV (10%, 2.046 ± 0.940%; 25%, 1.832 ± 0.486%; *t* = 0.709, *df* = 10, *P* = 0.495; Fig. 5*C*).

**Figure 6:**
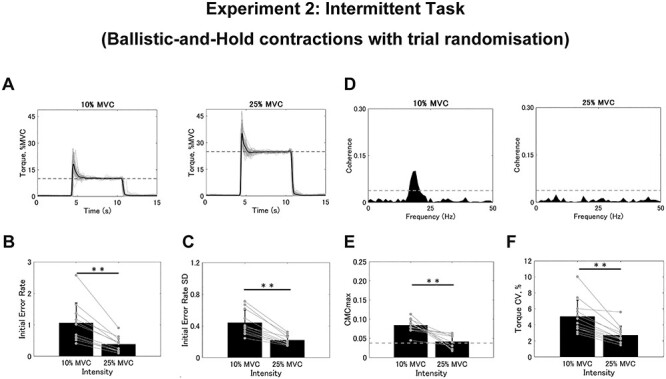
Results from the intermittent task in Experiment 2. (*A*) Typical examples of torque signals during the intermittent task. Gray plots represent the torque for the 60 trials and the black plot represents their average. Dashed lines represent the target intensities. (*B* and *C*) Group data for the individual mean initial error rate (*B*) and SD (*C*) calculated from 60 trials. (*D*) Typical examples of coherence spectra between EEG and rEMG for 10% and 25% of MVC. The dashed line represents the SL. (*E* and *F*) Group data for CMCmax (*E*) and torque CV (*F*). Black bars represent the average values for each contraction intensity. Black lines on these bars show SDs. The gray circles that are connected with a line represent the data points from each participant. Significant differences between the contraction intensities are denoted as follows: ^*^P < 0.05, ^*^^*^P < 0.01.

For the intermittent task, we examined the initial error again for both absolute and relative values. As represented in Figure 6*A*, the amount of initial error seems to be comparable between 10% and 25% of MVC. Indeed, the absolute value did not show significant differences between intensities (10%, 10.594 ± 6.340% MVC; 25%, 9.591 ± 6.122% MVC; *t* = 1.548, *df* = 10, *P* = 0.153), while this was not the case for the SD (10%, 4.405 ± 1.593% MVC; 25%, 5.472 ± 1.484% MVC; *t* = −4.668, *df* = 10, *P* = 0.001). As for the initial error rate, the values for 10% of MVC were again significantly larger than those for 25% of MVC in terms of both the initial error rate (10%, 1.059 ± 0.634; 25%, 0.384 ± 0.245; *t* = 5.440, *df* = 10, *P* < 0.001; Fig. 6*B*) and the SD (10%, 0.441 ± 0.159; 25%, 0.219 ± 0.059; *t* = 6.643, *df* = 10, *P* < 0.001; Fig. 6*C*), which supports the results of Experiment 1. Additionally, as represented in Figure 6*D*, the magnitude of CMC during the intermittent task varied remarkably between intensities such that it was greater in the trials with 10% versus 25% of MVC. When analyzed as group data, we observed significant differences in CMCmax (10%, 0.084 ± 0.018; 25%, 0.044 ± 0.016; *t* = 5.122, *df* = 10, *P* = 0.001; Fig. 6*E*) and CMCarea (10%, 0.334 ± 0.146; 25%, 0.065 ± 0.075; *t* = 6.362, *df* = 10, *P* < 0.001) such that these values were larger in the trials with 10% versus 25% of the MVC. Moreover, the torque CV was similarly larger in the trials with 10% than in 25% of MVC (10%, 4.578 ± 1.394%; 25%, 2.639 ± 1.127%; *t* = 6.516, *df* = 10, *P* < 0.001; Fig. 6*F*). Therefore, when the difference between the 2 intensities was magnified in Experiment 2, the findings were similar to those observed in Experiment 1.

### Experiment 3

**Figure 7:**
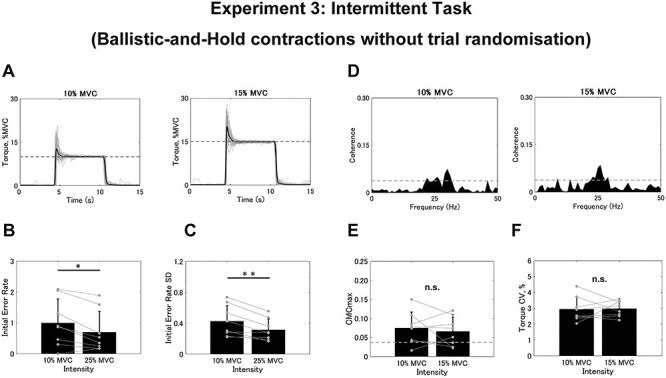
Results from the intermittent task in Experiment 3 without trial randomization. (*A*) Typical examples of torque signals during the intermittent task. Gray plots represent the torque for the 60 trials and the black plot represents their average. Dashed lines represent the target intensities. (*B* and *C*) Group data for the individual mean initial error rate (*B*) and SD (*C*) calculated from 60 trials. (*D*) Typical examples of coherence spectra between EEG and rEMG for 10% and 15% of MVC. The dashed line represents the SL. (*E* and *F*) Group data for CMCmax (*E*) and torque CV (*F*). Black bars represent the average values for each contraction intensity. Black lines on these bars show SDs. The gray circles that are connected with a line represent the data points from each participant. Significant differences between the contraction intensities are denoted as follows: ^*^P < 0.05, ^*^^*^P < 0.01. n.s. indicates no significance.

Eight out of the 13 participants who showed significant CMC were selected for further analyses in Experiment 3. All participants whose CMCmax exceeded the SL showed the peak frequency within the $\beta$-band (10%, 26.250 ± 4.500 Hz; 15%, 30.750 ± 3.304 Hz). In this experiment, we only administered the intermittent task to examine how the absence of randomization affected CMC. As represented in Figure 7*A*, the absolute initial error value was similar to that in Experiment 1 such that there was no significant differences between intensities (10%, 9.962 ± 7.760% MVC; 15%, 10.520 ± 10.051% MVC; *t* = −4.222, *df* = 7, *P* = 0.686), including the SD (10%, 4.264 ± 2.015% MVC; 15%, 4.731 ± 2.090% MVC; *t* = −2.030, *df* = 7, *P* = 0.082). In terms of the initial error rate, both the rate (10%, 0.996 ± 0.776; 15%, 0.701 ± 0.670; *t* = 3.169, *df* = 7, *P* = 0.016; Fig. 7*B*) and the SD (10%, 0.427 ± 0.202; 15%, 0.315 ± 0.139; *t* = 3.854, *df* = 7, *P* = 0.006; Fig. 7*C*) were significantly larger for in the trials with 10% versus 15% of MVC. Conversely, a representative example of the CMC spectra (Fig. 7*D*) shows no distinct difference between the intensities. Indeed, we found no significant differences between the 2 intensities for either CMCmax (10%, 0.075 ± 0.423; 15%, 0.066 ± 0.038; *t* = 0.621, *df* = 7, *P* = 0.555; Fig. 7*E*) or CMCarea (10%, 0.357 ± 0.348; 15%, 0.276 ± 0.378; *t* = 1.013, *df* = 7, *P* = 0.345). Additionally, we found no significant differences between intensities in terms of the torque CV (10%, 2.943 ± 0.775%; 15%, 2.969 ± 0.487%; *t* = −0.097, *df* = 7, *P* = 0.925; Fig. 7*F*). In summary, although the weaker intensity resulted in more deviation and a greater distribution of the initial error, as seen in Experiment 1, CMC itself was not significantly affected by contraction intensity.

### Experiment 4

**Figure 8:**
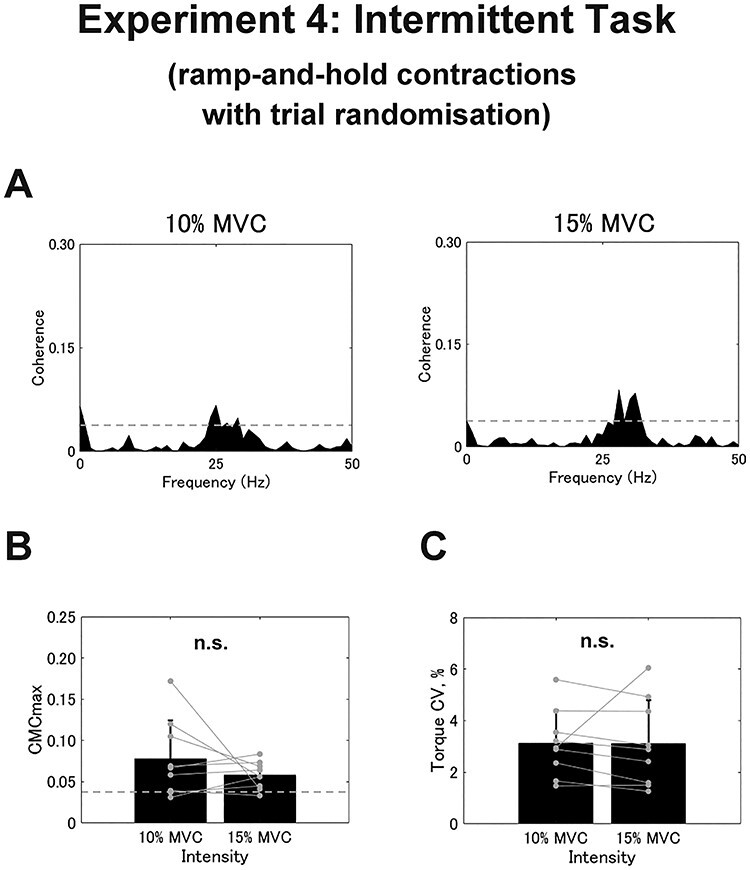
Results from the intermittent task in Experiment 4 performed with ramp-and-hold contractions. (*A*) Typical examples of coherence spectra between EEG and rEMG for 10% of MVC (*A*) and 15% of MVC. The dashed line represents the SL. (*B* and *C*) Group data for CMCmax (*B*) and torque CV (*C*). Black bars represent the average values for each contraction intensity. Black lines on these bars show SDs. The gray circles that are connected with a line represent the data points from each participant. n.s. indicates no significance.

Nine out of 12 participants showed significant CMC in Experiment 4. All participants whose CMCmax exceeded the SL showed the peak frequency within the $\beta$-band (10%, 23.500 ± 4.550 Hz; 15%, 25.333 ± 6.282 Hz). Since the intermittent task was carried out via ramp-and-hold contraction with gradual torque development, initial error values were not included in the analyses. As represented in Figure 8*A*, we found no significant difference between the 2 intensities for either CMCmax (10%, 0.078 ± 0.465; 15%, 0.058 ± 0.026; *t* = 1.180, *df* = 8, *P* = 0.272; Fig. 8*B*) or CMCarea (10%, 0.216 ± 0.246; 15%, 0.122 ± 0.091; *t* = 1.091, *df* = 8, *P* = 0.307). Further, the torque CV did not significantly differ according to the contraction intensity (10%, 3.118 ± 1.294%; 15%, 3.116 ± 1.671%; *t* = 0.006, *df* = 8, *P* = 0.996; Fig. 8*C*). In short, we did not observe any significant differences in CMCmax, CMCarea, or torque CV with respect to contraction intensity.

## Discussion

The present study examined the functional role of CMC on human motor control under consideration of the flow from motor initiation to maintenance. We first compared data from the conventional sustained task with that from the intermittent task in Experiment 1 and then performed 3 other experiments to examine the influence of different motor parameters. Through these experiments, we discovered that in a series of muscle contractions, including motor initiation and maintenance period, CMC is modulated in a context-dependent manner. Further, the magnitude of CMC is enhanced when active sensorimotor integration is required in feedback control periods because of the complexity and inaccuracy of preceding motor control.

### Corticomuscular Coupling Depends on Motor Requirements

During the sustained task in Experiments 1 and 2, we found no significant differences in CMC between contraction intensities. This is consistent with previous studies that reported that the magnitude of CMC was not altered by a weak-to-moderate level contraction (Brown et al. 1998; Mima et al. 1999; Ushiyama et al. 2012). However, during the intermittent task, a weaker contraction intensity (i.e., 10% of MVC) showed greater CMC. As CMC is considered to be a physiological indicator of sensorimotor integration (Baker and Baker 2003; Baker 2007; Witham et al. 2011), this implies that feedback control was enhanced during the intermittent task in trials with 10% of MVC in Experiments 1 and 2. Thus, the present data indicate that previous reports that CMC is not altered during contraction with a weak-to-moderate intensity range do not reflect universal phenomena, but instead, are context-dependent.

The observed significant difference in CMC between the 2 intensities in the intermittent task should be considered from a physiological perspective. In the sustained task, the difference in the contraction level could be simply generated by changing the number of recruited motor units because the spectral characteristics of EMG and CMC did not differ across contraction intensities. Conversely, the intermittent task in Experiments 1 and 2 resulted in differences in EMG amplitude and also modified CMC where a weaker intensity resulted in greater CMC. Therefore, in the intermittent task with ballistic-and-hold contractions, the motor output may have been modified not only by changing the number of recruited motor units but also by modulating corticomuscular coupling, which could determine the degree of synchrony of activated motor units.

The intermittent task required the participants to repeatedly develop torque as quickly as possible in every trial, which induced overshooting with respect to the target. During this motor initiation period, the trials with 10% of MVC resulted in a larger “initial error rate” (i.e., initial error size relative to the value of the target) and SD compared with those induced by the other contraction intensities in both Experiments 1 and 2. Unlike absolute values, which only reflect the size of the actual initial error, the relative initial error rate could be used to evaluate the accuracy of the initial motor output based on the specific contraction intensity. Our results indicate that trials with 10% of MVC would likely result in both larger relative initial error and greater intertrial variation. This suggests that the initial motor output based on the combination of feedforward and feedback control was less accurate and less stable for weaker contraction intensities.

When examining the fluctuation period during the intermittent task in Experiments 1 and 2, the CMC and torque CV were greater in trials with 10% of MVC compared with trials with the other contraction intensity. This result is consistent with previous reports that the magnitude of CMC was associated with force CV and $\beta$-band force PSD (Ushiyama et al. 2011a; Ushiyama et al. 2017). Here, when we consider both the CMC and torque CV, the increased CMC may reflect the extent of active error correction in the flow from motor initiation to maintenance. Additionally, as CMC involves neural oscillations, enhanced CMC may influence fluctuations in motor output. These fluctuations may be reflected in the torque CV, especially during contractions with a weak contraction intensity and large initial error. Therefore, we found increases in both CMC and torque CV in the trials with 10% of MVC, suggesting that oscillatory communication between the cortex and muscle is enhanced when active correction of the preceding motor error is required during the fluctuation period.

Unlike previous studies that adopted a sustained contraction task, our findings imply that the functional role of CMC could differ depending on what is required to perform each task with different motor contexts. The intermittent task used in the present study involved the emergence of initial error beforehand, which reflected the accuracy of motor output during the motor initiation period. Since the 2 intensities were presented in random order, the participants were not expected to repeat similar motor commands based on their experience in previous trials. Increased CMC might reflect active recalibration of the correspondence relation between somatosensory feedback and motor output, which is necessary to modify motor output with a large overshoot with respect to the target. However, conventional protocols that mainly focus on sustained contraction generally require participants to simply maintain constant motor output. In such situations, the observed CMC could reflect an “idling rhythm” that is necessary to achieve efficient motor control (Pfurtscheller et al. 1996; Kilner et al. 2000). Therefore, the functional role of CMC could vary according to motor tasks with different motor contexts.

Although we have interpreted the findings of Experiments 1 and 2 together, they were based on different research questions. The 2 intensities in Experiment 1, that is, 10% and 15% of MVC, were relatively close. This could have contaminated participant recognition of the difference between the contraction intensities, enhancing task difficulty. Conversely, the 2 intensities in Experiment 2, that is, 10% and 25% of MVC, were set to enable the participants to clearly recognize the difference. By conducting these 2 experiments, we were able to verify that a weak intensity was likely to result in large initial error rate and variability, leading to increased CMC and torque CV regardless of the difference between the 2 intensities. Thus, these experiments revealed that different motor control strategies were adopted for the 2 intensities in the intermittent task with ballistic-and-hold contractions under trial randomization. Specifically, in trials with weak contractions, inaccurate initial motor output could be compensated for by strengthening oscillatory corticomuscular coupling to facilitate sensorimotor integration.

### Trial Randomization as a Motor Context Modulating Corticomuscular Coupling

In Experiment 3, it is of interest to note that the difference in CMC between the 2 intensities (10% and 15% of MVC) vanished when we removed only the trial randomization. Thus, randomization appears to be a key motor context in modulating CMC during motor maintenance periods. To explore task-dependent differences, trial randomization has frequently been adopted in previous studies. However, our results imply that randomization itself could be a component of motor context that could intervene in the motor control strategy.

In Experiment 3, notably, the initial error rate and SD, but not the CMC or torque CV, were still significantly larger in the trials with 10% versus 15% of MVC. In other words, a weak contraction intensity tended to lead to unstable initial motor output, as in Experiments 1 and 2. Regarding this result, the contraction type (i.e., the intermittent task with ballistic-and-hold contractions) itself seemed to induce significant differences in the initial motor output according to contraction intensity, but it did not affect CMC in the absence of trial randomization. Therefore, the randomization in Experiments 1 and 2 may represent a crucial motor context that modulated the significant difference in CMC between the 2 contraction intensities.

Since the participants repeated contractions with the same intensity for 60 consecutive trials in Experiment 3, they could rely on a consistent motor output. Once they recognized the task requirement (i.e., the contraction intensity), they could simply repeatedly output motor commands at the same level and correct the initial error in the fluctuation period. Indeed, a computational study proposed that the repetition of similar trials leads to updating of the internal model to modify future motor planning (Shadmehr et al. 2010). This theory is in agreement with previous CMC studies that indicated while motor learning leads to increased CMC (Perez et al. 2006), it can be attenuated via motor adaptation (Kasuga et al. 2018). Thus, in Experiment 3, the participants may have habituated to the intermittent task for each of the contraction intensities such that the necessity of online sensorimotor recalibration did not differ between the 2 intensities. This situation might be similar to the sustained task in which the participants focused on just 1 contraction intensity, resulting in no significant differences in CMC between intensities.

Conversely, the intermittent task in Experiments 1 and 2 was more complicated, as it involved 2 intensities in the same set, presented with trial randomization. In such situations, the participants are required to frequently modulate their motor control strategy online. In particular, as the trials with 10% of MVC were conducted with inaccurate and unstable initial motor output, active sensorimotor integration may have been more required for error correction. To handle this situation, different oscillatory modes (i.e., stronger or weaker CMC modes) might be allocated to each contraction intensity. Furthermore, when more frequent and active error corrections were required, more attention and/or effort would be loaded on participants. Such difference in cognitive load depending on task difficulty could have also affected the magnitude of CMC (Kristeva-Feige et al. 2002; Watanabe et al. 2020).

In short, we suggest that the significant difference in CMC between the contraction intensities in the intermittent task in Experiments 1 and 2 was not determined by a single factor but by several motor contexts including contraction type, contraction intensity, and trial randomization. Yet, as the simplified task in Experiment 3 did not result in any significant differences in CMC, differentiation of the degree of sensorimotor integration between intensities does not appear to be necessary in the absence of randomization. Thus, we suggest that the magnitude of CMC is modulated according to complex and inaccurate motor initiation period in various motor contexts, necessitating active sensorimotor integration during the feedback control period.

### Effects of Limited Feedforward Control on Feedback Control Strategies

In Experiment 4 with intermittent ramp-and-hold contractions, we did not observe significant differences either in CMC or torque CV between the 2 contraction intensities (10% and 15% of MVC). During the motor task, the participants started exerting torque slowly in every trial. In such a situation, feedforward control would be limited, while feedback control was already engaged from the beginning of trials with no distinct overshoot or undershoot with respect to the target. As initial error correction was not urgently required in Experiment 4, we hypothesized that the participants would adopt a similar oscillatory feedback control strategy for the 2 contraction intensities in Experiment 4 in contrast to the behavior observed during the intermittent task in Experiments 1 and 2.

It was of interest to note that despite the trial randomization in Experiment 4, the magnitude of CMC did not differ between the 2 intensities. Since the participants started slow torque development with no necessity of active initial error correction, they could gradually adapt to the difference in the target for each trial. This would be a reason why trial randomization did not have a sufficiently critical impact to enable differentiation of CMC between contraction intensities. Instead, the CMC observed in this experiment could be thought of as an idling rhythm similar to the sustained tasks in Experiments 1 and 2 rather than the active sensorimotor integration for the correction of initial motor error due to the complexity and inaccuracy of preceding motor control.

### Technical Limitations of the Present Study

There are several potential limitations to our study. First, scalp EEGs cannot receive direct activities from cortical neurones because of the presence of buffers such as the skull and cerebrospinal fluid (Malmivuo et al. 1997; Mima et al. 1999; Grosse et al. 2003). This issue also affects recording using surface EMGs, which can be affected by individual fat, skin tissue, or muscle anatomy (Farina et al. 2002). In light of these points, we were not able to obtain uniform signals among the participants. However, CMC is determined by the constancy of the amplitude ratio and phase difference between 2 signals (Halliday et al. 1995). As the abovementioned factors may modulate the amplitudes of signals, we do not expect that they had a major impact on the CMC analyses in the present study.

Second, we only obtained EEGs from 5 scalp positions over the sensorimotor cortex representing the foot. Although this could be considered a small number of channels, we expected it to be sufficient for examining CMC between the lower limb muscles and associated representation area. However, if we had used whole brain EEG recording, we could have also investigated interconnections across cortical areas to examine the functional connectivity related to the context-dependent motor control. For instance, the participants in the present study were also depending on visual information to detect motor errors. Therefore, information about intercortical communication across visual, somatosensory, and motor areas could be valuable in terms of the neural mechanisms that support feedforward and feedback control of sequential movements.

Third, in addition to CMC, coherence between the spinal cord and muscles (i.e., spinomuscular coherence) has been observed during isometric contractions in monkeys (Takei and Seki 2008; Oya et al. 2020). Briefly, an increase in spinomuscular coherence was reported during the grip phase (i.e., force development stage) of a motor task, whereas CMC was increased in the hold phase (i.e., sustained contraction). Thus, if we had incorporated a spinomuscular coherence examination in this study, we could have more comprehensively considered context-dependent motor control. Because of the limitations associated with human studies, we were only able to evaluate CMC as a marker of neural control in feedback control of movements. Still, CMC studies in humans are advantageous in that the experimenter can verbally explain the task requirement to the participants and present several motor tasks with different motor parameters. This enables the examination of various motor control strategies with different motor task designs. Therefore, we believe that the obtained context-dependency of CMC reflects a unique aspect of physiological studies in humans.

Apart from the abovementioned limitations, we rejected participants who did not show significant CMC from further analyses. However, even when CMC could not be observed from the obtained signals, it does not mean that these participants do not have neural communication between the motor cortex and periphery. As the magnitude of CMC differs among individuals (Ushiyama et al. 2011b), a percentage of the population are likely to exhibit little or no significant CMC. It should be noted that no significant CMC does not necessarily mean the lack of neural communication between the motor cortex and the periphery. Although we focused on CMC as a key measure in the present study, further investigation with other indicators will be needed to include participants with no significant CMC.

## Conclusion

In the present study, we found that in a series of intermittent ballistic-and-hold contractions with 2 contraction intensities under trial randomization, the magnitude of CMC was significantly greater for weaker intensity contractions when the initial motor output had larger deviations and variations. These data indicate that trials with inaccurate initial motor output were handled via enhanced synchrony between the sensorimotor cortex and muscle during the feedback control period. Furthermore, these differences in CMC disappeared when the trials were nonrandomized or when the torque development speed was slower. Thus, we suggest that CMC, as a physiological indicator of feedback control, is modulated context-dependently during a series of movements. Further, its magnitude would be enhanced when active sensorimotor integration is required in the feedback control period due to the complexity and inaccuracy of preceding motor control.

## Notes

We thank Ms Tomomi Hamaoka, Ms Kana Iijima, and Ms Chieko Matsuda for their secretarial assistance. We also thank Sydney Koke, MFA, from Edanz Group (https://en-author-services.edanzgroup.com/ac) for editing a draft of this manuscript. R.S. and J.U. conceptualized and designed the study, interpreted data, and wrote the manuscript. R.S. acquired and analyzed data. J.U. supervised the study and acquired funding. *Conflict of Interest*: None declared.

## Funding

Grant-in-Aid for Scientific Research (B) (Japan Society for the Promotion of Science, JSPS) (20H04091 to J.U.); Grant-in-Aid for Challenging Exploratory Research (JSPS) (16K12971 to J.U.); Sasakawa Scientific Research Grant (The Japan Science Society, JSS) (2020-6005 to R.S.); Living Platform, Ltd., Japan.
